# Modeling the oxygen transport to the myocardium at maximal exercise at high altitude

**DOI:** 10.14814/phy2.15262

**Published:** 2022-04-19

**Authors:** Jean‐Paul Richalet, Eric Hermand

**Affiliations:** ^1^ 27097 UMR INSERM U1272 Hypoxie & Poumon Université Sorbonne Paris Nord Bobigny France; ^2^ Université Littoral Côte d’Opale Université Artois Université Lille, CHU Lille ULR 7369 ‐ URePSSS‐Unité de Recherche Pluridisciplinaire Sport Santé Société Dunkerque France

**Keywords:** autonomic nervous system, exercise, heart, high intensity, hypoxia, myocardium, oxygen pressure and saturation

## Abstract

Exposure to high altitude induces a decrease in oxygen pressure and saturation in the arterial blood, which is aggravated by exercise. Heart rate (HR) at maximal exercise decreases when altitude increases in prolonged exposure to hypoxia. We developed a simple model of myocardial oxygenation in order to demonstrate that the observed blunting of maximal HR at high altitude is necessary for the maintenance of a normal myocardial oxygenation. Using data from the available scientific literature, we estimated the myocardial venous oxygen pressure and saturation at maximal exercise in two conditions: (1) with actual values of maximal HR (decreasing with altitude); (2) with sea‐level values of maximal heart rate, whatever the altitude (no change in HR). We demonstrated that, in the absence of autoregulation of maximal HR, myocardial tissue oxygenation would be incompatible with life above 6200 m–7600 m, depending on the hypothesis concerning a possible increase in coronary reserve (increase in coronary blood flow at exercise). The decrease in maximal HR at high altitude could be explained by several biological mechanisms involving the autonomic nervous system and its receptors on myocytes. These experimental and clinical observations support the hypothesis that there exists an integrated system at the cellular level, which protects the myocardium from a hazardous disequilibrium between O_2_ supply and O_2_ consumption at high altitude.

## Graphical abstract

Maximal heart rate (HR) is known to be lower at high altitude than at sea level, but the underlying mechanisms are still to be fully explained. From field data extracted from subjects exercising at maximal intensity above 4000 m, our model shows that an unchanged HR at high altitude would imply myocardial oxygenation inadequate for functioning and survival. Hence, local mechanisms, relying on the regulation of the autonomic nervous system, would play a key role in preventing myocardium from a potentially harmful high metabolic demand. This is a remarkable example of autoregulation of a vital organ facing extreme constraints.

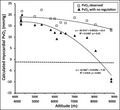

## 1 INTRODUCTION

Exposure to high altitude induces a decrease in oxygen pressure along the gradient from ambient air to cell mitochondria. The degree of hypoxemia is aggravated by the progressive decrease in atmospheric PO_2_ so that the severity of tissue hypoxia increases with altitude. Physical exercise is a potent factor that aggravates the level of hypoxemia since, at high altitude, arterial PO_2_ decreases when the intensity of exercise increases (West et al., 1983). This phenomenon has been clearly linked, at least in part, to a diffusion limitation in the lungs (Wagner, 2010; West et al., 1983). Two factors may be responsible for this limitation: (1) cardiac output increases with exercise intensity, causing a decrease of blood transit time in the pulmonary capillaries, hence reducing the time required for oxygen diffusion through the alveolo‐capillary barrier; (2) due to a lower arterial O_2_ content, peripheral O_2_ extraction increases and PO_2_ in the venous blood coming back to the lungs is lowered, rending a proper reloading of O_2_ in the capillaries more difficult (Mollard et al., 2007; Van Thienen & Hespel, 2016).

The myocardium is very sensitive to O_2_ availability, especially when energetic demand is high such as during exercise. Therefore, the myocardium is submitted to a high constraint in terms of O_2_ availability when exposed to both hypoxia and intense exercise. In this matter, if the maximal work of myocardium depends on mitochondrial O_2_ content, the latter itself follows the variation of venous PO_2_ (Gnaiger et al., 1995; Sutton et al., 1988), so we could assume that myocardial venous PO_2_ is a valuable index of cardiac O_2_ consumption, even if it is not likely to be linear.

Paradoxically, in alpinists exercising in extreme conditions over the altitude of 8000 m with an arterial PO_2_ of around 35 mmHg, no cardiac failure, coronary insufficiency, angina pectoris or myocardial infarct has ever been reported (Mallet et al., 2021; Reeves et al., 1987). In parallel, heart rate at high altitude, although increasing at submaximal exercise for any level of workload, is greatly reduced at maximal exercise (Richalet, 2016), hereby protecting the myocardium against a too high energy consumption in conditions of low O_2_ availability. An important series of studies in animals and humans have been performed to explain this decrease in maximal heart rate and developed the hypothesis of a downregulation of beta‐adrenergic receptors in the myocardium in prolonged exposure to hypoxia, together with an increase in parasympathetic influence (Antezana et al., 1994; Boushel et al., 2001; Favret & Richalet, 2007; Favret et al., 2001; Hartley et al., 1974; Kacimi et al., 1993; León‐Velarde et al., 2001; Richalet, Mehdioui, et al., 1988; Siebenmann et al., 2017; Voelkel et al., 1981). This modulation of cardiac receptors would reduce the chronotropic response to the hypoxia‐induced adrenergic activation and protect the myocardium in these extreme conditions (Richalet, 2016).

The present study aims to develop a model of O_2_ transport in the myocardium at exercise in hypoxia in acclimatized subjects in order to demonstrate that the decrease in maximal heart rate at high altitude is necessary for the survival of myocardial tissue in these extreme conditions.

## 2 MATERIAL AND METHODS

### 2.1 Model description

**FIGURE 1:**
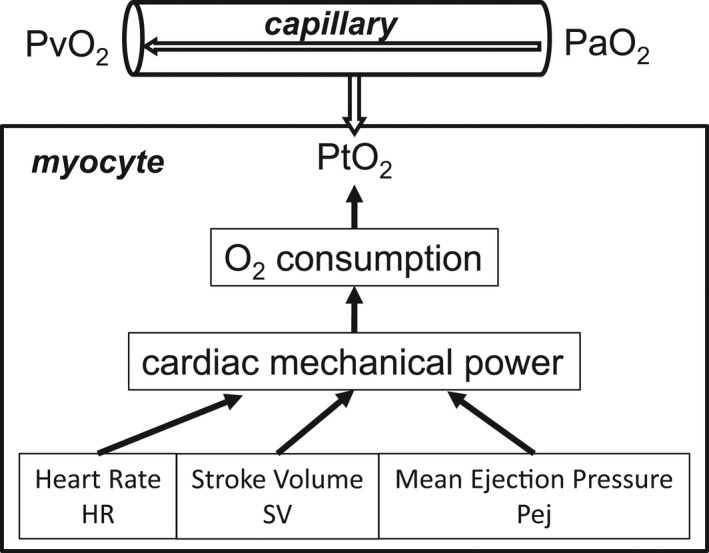
Model of oxygen handling in the myocyte. Tissue PO_2_ (PtO_2_) depends on the balance between O_2_ availability after diffusion from the capillary and O_2_ consumption by the myocyte. O_2_ consumption is determined by cardiac mechanical power, which mainly depends on three factors: Heart rate (HR), stroke volume (SV), and mean ejection pressure (Pej)

Monitoring the level of oxygenation of the myocardial tissue would require measuring PO_2_ within the tissue, which is not readily feasible in humans exercising in altitude conditions. Therefore, we aimed to determine an alternative method that would give us an indirect measure of tissue and mitochondrial oxygenation, represented by myocardial venous blood PO_2_. A model of O_2_ transport to the myocardium is given in Figure 1. Along the myocardial capillary, blood PO_2_ is progressively decreasing from the arterial to the venous end while O_2_ is diffusing to the tissue. We can assume that end‐capillary PO_2_ is in equilibrium with tissue PO_2_, therefore, venous PO_2_, equal to end‐capillary PO_2_, would be a reliable substitute to tissue PO_2_ (Gnaiger et al., 1995; Herrmann & Feigl, 1992; Rubio & Berne, 1975; Sutton et al., 1988). The objective is therefore to calculate myocardial venous PO_2_, a marker of myocardial tissue oxygenation, as a function of altitude in the condition of maximal exercise.

### 2.2 Determinants of myocardial tissue PO_2_

Myocardial tissue PO_2_ is the result of O_2_ consumption and O_2_ availability. Oxygen consumption is determined by the cardiac mechanical power of the left and right ventricles $\left({\dot{W}}_{\text{LV}}\;\text{and}\;{\dot{W}}_{\text{RV}}\right)$, which depends on heart rate (HR), stroke volume (SV), and mean ejection pressure of each ventricle, in the aorta and in the pulmonary artery (PejAo and PejPa, respectively) (Opie, 1991):

${\dot{W}}_{\text{LV}}=\text{HR}\times \text{SV}\times \text{PejAo}$ and ${\dot{W}}_{\text{RV}}=\text{HR}\times \text{SV}\times \text{PejPa}$

Myocardial O_2_ consumption $\left({\dot{V}O}_{2}\right)$ is linked to cardiac mechanical power by the energetic equivalent of O_2_ for the myocardium EE (Han et al., 2019):

(1) $$\begin{array}{c} \dot{V}O_{2}=\text{EE}\times \left({\dot{W}}_{\text{LV}}+{\dot{W}}_{\text{RV}}\right)=\text{EE}\times \text{HR}\times \text{SV}\times \left(\text{PejAo}+\text{PejPa}\right) \end{array}$$

From the O_2_ transport side, O_2_ consumption can be derived from myocardial blood flow $\left(\dot{Q}\right)$ and myocardial arterio‐venous difference in O_2_ content (Ca–Cv), using the Fick equation:

$$\overset{˙}{V}O_{2}=\dot{Q}\times \left(\text{Ca}‐\text{Cv}\right)$$

or

(2) $$\begin{array}{c} \dot{V}O_{2}=\dot{Q}\times 1.34\times \left[\text{Hb}\right]\times \left({\text{SaO}}_{2}‐{\text{SvO}}_{2}\right) \end{array}$$

where [Hb] is the blood concentration of hemoglobin, SaO_2_ and SvO_2_ are the O_2_ saturation in the arterial and myocardial venous blood, respectively.

Combining Equations (1) and (2), it comes:

$$\begin{array}{c} \begin{array}{cc} \dot{V}O_{2} & \;=\text{EE}\times \text{HR}\times \text{SV}\times \left(\text{PejAo}+\text{PejPa}\right)\\ & \;=\dot{Q}\times 1.34\times \left[\text{Hb}\right]\times \left({\text{SaO}}_{2}‐{\text{SvO}}_{2}\right) \end{array} \end{array}$$

This equation can be rewritten as follows:

(3) $$\begin{array}{c} \text{HR}=\dot{Q}\times \left[\text{Hb}\right]\times \left({\text{SaO}}_{2}‐{\text{SvO}}_{2}\right)\times A \end{array}$$

where

$$A=\frac{1.34}{\text{EE}\times \text{SV}\times \left(\text{PejAo}\;+\;\text{PejPa}\right)}$$

Let us write this equation for heart rate at maximal exercise in normoxic (mn) and hypoxic (mh) conditions:

$$\text{HRmn}=\dot{Q}\text{mn}\times \left[\text{Hb}\right]\text{mn}\times \left(\text{Samn}‐\text{Svmn}\right)\times \text{Amn}$$

$$\text{HRmh}=\dot{Q}\text{mh}\times \left[\text{Hb}\right]\text{mh}\times \left(\text{Samh}‐\text{Svmh}\right)\times \text{Amh}$$

and the ratio $\frac{\text{HRmh}}{\text{HRmn}}$:

(4) $$\begin{array}{c} \frac{\text{HRmh}}{\text{HRmn}}=\frac{\dot{Q}\text{mh}}{\dot{Q}\text{mn}}\times \frac{\left[\text{Hb}\right]\text{mh}}{\left[\text{Hb}\right]\text{mn}}\times \frac{\text{Samh}‐\text{Svmh}}{\text{Samn}‐\text{Svmn}}\times \frac{\text{Amh}}{\text{Amn}} \end{array}$$

In order to estimate HRmh as a function of HRmn, we need to evaluate the changes induced by hypoxia in the above ratios in Equation (4).

First, the ratio $\frac{\dot{Q}\text{mh}}{\dot{Q}\text{mn}}$ is the ratio of myocardial blood flow at maximal exercise between normoxia and hypoxia, for example, the “coronary reserve” that can be mobilized in hypoxia. Although there is no data in the literature above 4500 m, it is likely that coronary reserve is near maximal in normoxia and can hardly increase in hypoxia (Wyss et al., 2003). Therefore, this ratio is close to unity. In a second part of the study, we will evaluate the possible influence of a substantial increase in coronary reserve (see below).

Second, the ratio $\frac{\left[\text{Hb}\right]\text{mh}}{\left[\text{Hb}\right]\text{mn}}$ represents the intensity of the erythropoiesis induced by the prolonged exposure to high altitude. It is 1 in acute hypoxia and increases with acclimatization: For example, if [Hb] is 15 g/dl in normoxia and goes up to 20 g/dl in prolonged hypoxia, this ratio will be 1.33.

Third, the ratio $\frac{\text{Samh}‐\text{Svmh}}{\text{Samn}‐\text{Svmn}}$ represents the change in arterio‐venous difference in O_2_ saturation at maximal exercise from normoxia to hypoxia. We know from the literature that Samn is normally around 98% and that Svmn is around 30%, so that the arterio‐venous difference in saturation in normoxia is around 68% (Heiss et al., 1976; Richalet et al., 1981). Altitude‐induced changes in arterial O_2_ saturation at maximal exercise are known from the literature. However, myocardial venous O_2_ saturation at maximal exercise (Svmh) has never been measured yet.

Finally, the ratio $\frac{\text{Amh}}{\text{Amn}}$ depends on the ratio of energetic equivalents, the ratio of stroke volumes and the ratio of ejection pressures. Although no data is available, the energetic equivalent is probably not modified by altitude, unless profound changes in substrate utilization occur in hypoxia. Stroke volume is marginally modified in hypoxia: while a 10% decrease has been measured at rest, its value at maximal exercise at altitude (7620 m) has been estimated at 86% of its sea level value (Reeves et al., 1987; Sutton et al., 1988). Mean aortic pressure at exercise does not consistently increase at high altitude, while mean pulmonary pressure increases through pulmonary vasoconstriction (Boussuges et al., 2000). The sum of mean aortic + pulmonary pressures has been estimated to go from 153 mmHg at sea level to 150, 169 and 157 mmHg at 6100 m, 7620 m and 8840 m, respectively (Sutton et al., 1988). Altogether, the ratio $\frac{\text{Amh}}{\text{Amn}}$ probably stays around the unity since a decrease in stroke volume would compensate an increase in ejection pressures (Stembridge et al., 2016; Sutton et al., 1988).

Finally, if we summarize our first assumptions (no change in coronary reserve and compensations in variations of ejection volumes and pressures), we can write that:

(5) $$\frac{\dot{Q}\text{mh}}{\dot{Q}\text{mn}}\times \frac{\text{Amh}}{\text{Amn}}=1$$

Therefore, combining Equations (4) and (5):

$$\frac{\text{HRmh}}{\text{HRmn}}=\frac{\left[\text{Hb}\right]\text{mh}}{\left[\text{Hb}\right]\text{mn}}\times \frac{\text{Samh}‐\text{Svmh}}{\text{Samn}‐\text{Svmn}}$$

Estimating Samn‐Svmn at 68% (see above), we can calculate Svmh as a function of Samh as follows:

(6) $$\text{Svmh}=\text{Samh}‐68\times \frac{\text{HRmh}\;\times \left[\text{Hb}\right]\text{mn}}{\text{HRmn}\times \left[\text{Hb}\right]\text{mh}}$$

**FIGURE 2:**
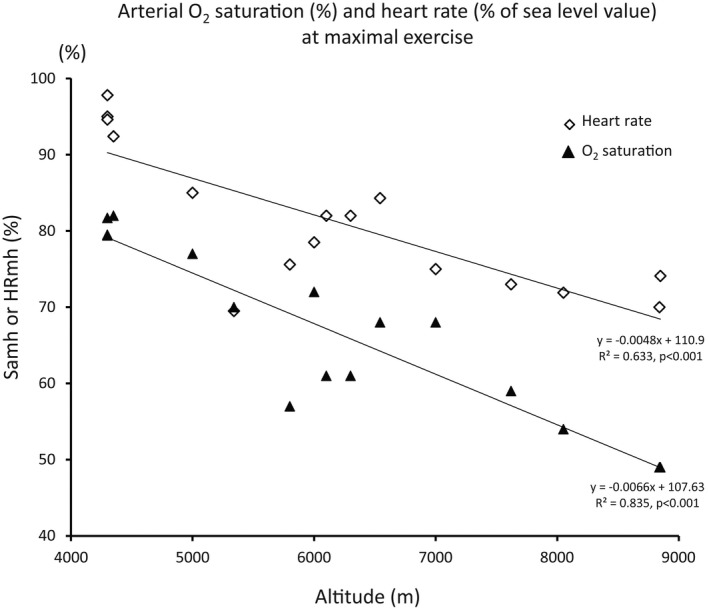
Values of arterial O_2_ saturation at maximal exercise (Samh) and its corresponding HR (expressed as the percentage of maximal HR at sea level), in subjects acclimated to hypoxia, as a function of altitude. Data extracted from literature (references in Table 1). There is a significant linear decrease in Samh and in HRmh with increasing altitude

Samh can be estimated by linear regression from our data (Table1, Figure 2) by the following equation:

(7) $$\text{Samh}=107.6‐0.0066\times \text{Altitude}\left(m\right)$$

**TABLE 1:** Data from the literature was used to build the model of oxygen transport in the myocardium at maximal exercise in hypoxia

Reference	Altitude (m)	$\frac{\mathrm{HRmh}\;}{\mathrm{HRmn}}$	Samh	$\frac{[\mathrm{Hb}]\mathrm{mh}\;}{[\mathrm{Hb}]\mathrm{mn}}$	Nb days
Moore et al. (1986)	4350	0.924	82	1.23	19
Pugh et al. (1964)	4600	0.928			30–90
	5800	0.756	57	1.47	60–90
Klausen et al. (1966)	3800	0.89			25
	4340	0.906			16
Vogel et al. (1967)	4300	0.978	79.4	1.07	3
	4300	0.95	81.7	1.11	17
Dill & Adams (1971)	3090	0.944			17
Vogel et al. (1974)	4350	0.924			10
Cerretelli (1976)	5350	0.87		1.37	
Horstman et al. (1980)	4300	0.963			15
Saltin et al. (1968)	4300	0.946	79.5	1.13	15
Dill et al. (1969); Klausen et al. (1970)	3800	0.899		1.08	20
Vogel et al. (1974)	4600	0.873			3
Sutton et al. (1988)	6100	0.82	61	1.2	
	7620	0.73	59	1.26	
	8840	0.70	49	1.26	
Christensen & Forbes (1937)	5340	0.695	70	1.5	9–10
Richalet (1983)	5000	0.859			21
Richalet et al. (1988)	4350	0.952			8
	4800	0.901	92		21
West et al. (1983); Winslow et al. (1984)	6300	0.82	61	1.29	
	8050	0.719	57	1.27	
	8848	0.741	49	1.29	
Young et al. (1982)	4300	0.874			15
Antezana et al. (1994)	6542	0.843	68	1.13	7
Richalet et al. (1999); Robach et al. (2000)	5000	0.85	77	1.1	2–6
	6000	0.785	72	1.07	9–12
	7000	0.75	68	1.14	15–19

$\frac{\text{HRmh}\;}{\text{HRmn}}$: ratio of maximal heart rate measured at high altitude over value measured at sea level; Samh, arterial O_2_ saturation at maximal exercise at high altitude; $\frac{\left[\text{Hb}\right]\text{mh}\;}{\left[\text{Hb}\right]\text{mn}}$, ratio of hemoglobin concentration measured at high altitude over value measured at sea level; nb days, number of days spent at high altitude.

Equation (6) then allows calculating myocardial venous O_2_ saturation at maximal exercise in various altitude conditions if arterial O_2_ saturation, heart rate, and hemoglobin concentrations are known. From O_2_ saturation (SO_2_), we can estimate O_2_ pressure (PO_2_), given a standard equation of the oxyhemoglobin dissociation curve and an estimated value of venous pH of 7.32:

$${\text{PO}}_{2}=\frac{29.11\times {\text{SO}}_{2}}{{\left(100‐{\text{SO}}_{2}\right)}^{0.3704}}$$

Dash et al.(2016) Therefore, we reach our main objective: estimating venous tissue O_2_ pressure at maximal exercise at various altitudes and evaluating the influence of maximal heart rate on tissue oxygenation.

### 2.3 Summary of main assumptions

In order to build the present model, we made several assumptions, as follows:

- There is no significant increase in coronary reserve at high altitude (in a first approach).
- Arterio‐venous difference in oxygen saturation in normoxia equals 68%.
- Decrease in stroke volume in hypoxia compensates an increase in ejection pressures.
- Coronary venous pH at exercise is 7.32.

### 2.4 Data from the literature

In order to feed our model, we reviewed all available studies in the literature that simultaneously proposed values of heart rate, hemoglobin concentration, and arterial O_2_ saturation for various altitudes above 4000 m at maximal exercise. Data from studies concerning prolonged exposure to hypoxia (>3 days) were included and studies concerning acute hypoxia were excluded. The first historical values come from the “International High Altitude Expedition to Chile” in 1935 (Christensen & Forbes, 1937). Values are presented in Table 1.

### 2.5 Role of coronary reserve

Very few studies are available about coronary reserve at maximal exercise, especially at high altitude. Wyss and coworkers found no significant increase in acute hypoxia (4500 m) (Wyss et al., 2003). However, studies by Kaufmann and coll. have shown that it may increase by 20% at 4559 m (Kaufmann et al., 2008). To our knowledge, no value is available at higher altitudes. However, we evaluated how our model is modified, assuming that coronary reserve at maximal exercise may increase from sea level to high altitude. If we suppose that the minimal value of myocardial venous O_2_ saturation compatible with adequate O_2_ supply to the myocardium is 10% (Goodwill et al., 2017), we can calculate from Equations (4) and (7) the maximal altitude (maxAlt) compatible with this minimal O_2_ saturation as a function of an estimated percentage increase in coronary reserve at maximal exercise (ΔQhn) from sea level to a given altitude:

(8) $$\text{maxAlt}\;=\;14788\;‐\;\frac{8445}{\left(1+\frac{\Delta \text{Qhn}}{100}\right)}$$

## 3 RESULTS

Using equation (6) and Table 1, we can calculate Svmh in two scenarios:

1. Using the actual value of HRmh observed in the studies quoted in Table 1
2. Considering that there is no decrease in HRmh at altitude, so that the ratio $\frac{\text{HRmh}}{\text{HRmn}}$ is 1.

**FIGURE 3:**
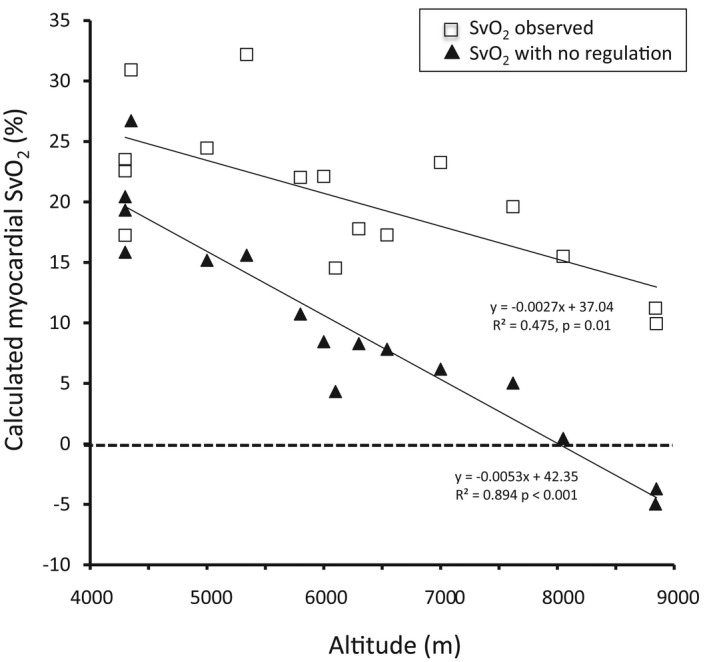
Calculated values of myocardial venous O_2_ saturation (SvO_2_) at maximal exercise as a function of altitude in prolonged exposure to hypoxia. In open squares, values are calculated using data from the literature (Table 1) with the actual value of maximal heart rate (decreasing from sea level). In black triangles, values are re‐calculated using the same data but with a value of maximal heart rate at altitude identical to the sea‐level value. Note that with the actual values, SvO_2_ stays over 10% (minimal value compatible with normal myocardial oxygenation), while if we suppose that maximal heart rate does not decrease with altitude, SvO_2_ plunges below 10% over 6200 m and becomes negative above 8000 m, values incompatible with life. Negative values of SvO_2_ are physiologically impossible in the case of the absence of regulation

Results are shown in Figure 3.

**FIGURE 4:**
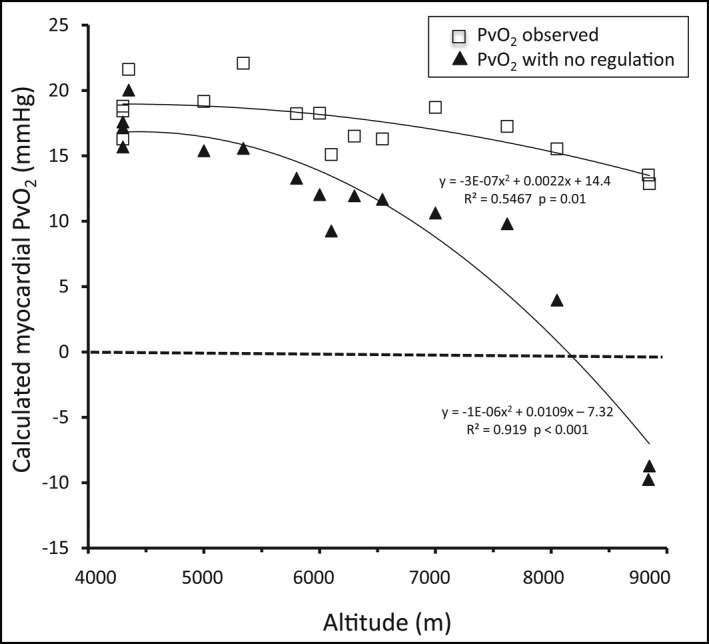
Calculated values of myocardial venous PO_2_ (PvO_2_) in the same conditions as in Figure 3. Pv at maximal exercise stays almost constant, whatever the altitude, thanks to the autoregulation of maximal heart rate (see text for explanations). Negative values of PvO2 are physiologically impossible in the case of the absence of regulation

Considering the second hypothesis of no decrease in maximal heart rate at altitude, venous O_2_ saturation decreases with altitude and becomes negative above 8000 m, condition that is not physiologically compatible with life. Similarly, values of venous PO_2_ become negative around 8000 m (Figure 4).

In contrast, taking the first hypothesis, there is only a slight decrease in venous saturation and pressure but not as pronounced as for the first hypothesis (Figures 3 and 4).

**FIGURE 5:**
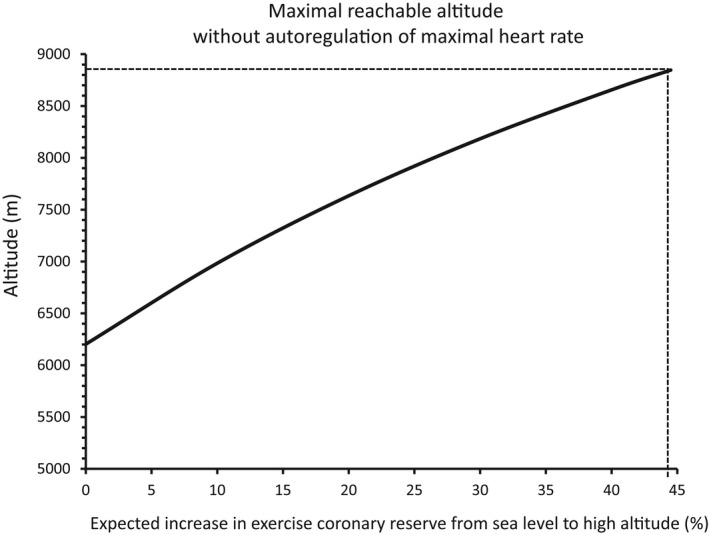
Maximal reachable altitude compatible with normal myocardial oxygenation (myocardial venous O_2_ saturation above 10%) as a function of an expected increase in coronary reserve at maximal exercise from sea level to high altitude, if we suppose that maximal heart rate does not decrease with altitude (no autoregulation). Note that if we consider that coronary reserve at maximal exercise is already maximal at sea level, the maximal tolerated altitude would be 6200 m. If we hypothesize a 20% increase in coronary reserve, the maximal altitude would be 7600 m. To reach the summit of Mount Everest (8848 m), the coronary reserve would have to increase by 44.5%

Figure 5 shows that if we suppose that coronary reserve at maximal exercise is already maximal at sea level, the maximal reachable altitude compatible with myocardial euoxia is around 6200 m in case of no regulation of maximal heart rate. To reach the summit of Mount Everest without decrease in maximal heart rate, the increase in coronary reserve would have to be as high as 44.5%.

## 4 DISCUSSION

The present model was constructed from the physiological data available in the literature. However, as expected, very few measurements are available in humans in those extreme conditions of exercise and altitude, so that we had to make some reasonable assumptions. To reduce the uncertainty of these assumptions, future studies may include measurements of myocardial blood flow, cardiac venous and mitochondrial PO_2_ at maximal exercise, both at sea level and high altitude. Let us reconsider the above assumptions and estimate the effects on the results of a non‐validity of some of them.

First, arterial hypoxemia is a probably the most powerful stimulus for coronary vasodilation, either directly or through active metabolites such as adenosine, NO or prostaglandins. However, hypoxia‐induced vasodilation is limited (coronary reserve). If myocardial blood flow at maximal exercise can increase significantly at high altitude, let us suppose that the maximal value of ratio $\frac{\dot{Q}\text{mh}}{\dot{Q}\text{mn}}$ is 1.2 (20% increase), as previously suggested (Kaufmann et al., 2008). In that condition, maximal altitude reachable would be around 7600 m (Figure 5). The minimal value of this ratio suitable to reach the summit of Mount Everest (8848 m) would be 44.5%, which is incompatible with our present understanding of the regulation of myocardial blood flow and adequate myocardial oxygenation.

Second, if the increase in ejection pressures largely overpasses the decrease in stroke volume, the conditions would be worse for myocardial oxygenation, as inferred by Equation (4). Conversely, if pressures do not change and stroke volume largely decreases, conditions of oxygenation would be better, but this hypothesis is incompatible with values of ejection pressures and volumes available in the literature (Naeije, 2010; Stembridge et al., 2016; Sutton et al., 1988).

From the present modeling study, based on measured values from the literature, we suggest that the hypothesis of a preservation of maximal heart rate at high altitude at its sea level value would necessarily lead to values of myocardial tissue PO_2_ incompatible with a viable myocardial oxygenation. Therefore, the alternative hypothesis of a mechanism limiting heart rate at exercise in hypoxic conditions therefore appears realistic (Figure 2). We hypothesize that cardiac chronotropic function could be controlled by a local mechanism linked to myocardial PO_2_ (White et al., 1995). Several pathways have been mentioned in the literature. A downregulation of the adrenergic system has been shown in prolonged hypoxia, either in humans or animal models (Favret & Richalet, 2007). Adrenergic activation is well documented in acute and prolonged hypoxia (Antezana et al., 1994; Richalet et al., 1990) but the response to this activation is blunted as shown by a lower heart rate for a given value of plasma norepinephrine at exercise (Antezana et al., 1994; Richalet, Mehdioui, et al., 1988) or for a given value of perfused isoproterenol (Richalet, Larmignat, et al., 1988). In parallel, although a chronic exposure to 3500 m triggers a long‐term reduction of the vagal tone at rest (Ponchia et al., 1994; Siebenmann et al., 2017), the parasympathetic system may be activated as shown by the restoration of heart rate at exercise after infusion of a muscarinic blocker (Bogaard et al., 2002; Boushel et al., 2001; Hartley et al., 1974). In a model of rats exposed to prolonged hypoxia, the density of beta‐adrenergic receptors has been shown decreased, while, conversely, the density of muscarinic receptors is increased (Kacimi et al., 1992, 1993; Voelkel et al., 1981). The complex pathway connecting adrenergic, muscarinic, and adenosinergic receptors to the adenylate cyclase in the cardiomyocyte is modified when exposed to hypoxia: the activity of the Gs protein is reduced while the expression of Gi protein is enhanced, both phenomenon leading to a blunting of adenylate cyclase activity and a reduced chronotropic function (Favret & Richalet, 2007; Fowler et al., 1986; Kacimi et al., 1995; León‐Velarde et al., 2001; White et al., 1995). Moreover, an extensive evidence exists concerning the role of downregulation of adrenergic receptors in cardiac failure, another representative condition of imbalance between cardiac oxygen supply and consumption (Hamdani & Linke, 2012; Soltysinska et al., 2011). The heart is not the only organ where these desensitization mechanisms appear in hypoxia. Fat cells also show a decrease in their response to adrenergic activation in prolonged hypoxia (de Glisezinski et al., 1999). Renal handling of calcium is submitted to a down‐regulation of parathormone effects in hypoxia (Souberbielle et al., 1995). Similarly, growth hormone production is subjected to a down‐regulation of its specific receptor (Richalet et al., 2010). Lactate release by the muscle could be modulated by a down‐regulation of beta‐receptors (Reeves et al., 1992). Common elements in all these signaling pathways seem to be receptors regulated by a G protein complex (Hamdani & Linke, 2012; Richalet, 2016).

## 5 CONCLUSION

Altogether, there appears to exist an integrated system at the cellular level that protects the myocardium from a hazardous disequilibrium between O_2_ supply and O_2_ consumption at high altitude. This system would fully explain the decrease in heart rate at maximal exercise at high altitude. This autoregulation of O_2_ supply in the myocardium efficiently protects this vital organ against myocardial ischemia and its potentially serious clinical consequences (Richalet, 1997, 2016). Simple modeling of biological mechanisms may help for a better understanding of regulation systems in complex environmental conditions. This paper allows some significant advances in the knowledge of physiological adaptations to stressors such as hypoxia. It is a remarkable example of autoregulation of a vital organ submitted to a severe metabolic challenge that contributes to an overall process of homeodynamics (Hermand et al., 2021; Richalet, 2021). Future studies may include measurements of myocardial blood flow, cardiac venous, and mitochondrial PO_2_ at maximal exercise, both at sea level and high altitude, to validate and refine our model.

## CONFLICTS OF INTEREST

None.

## AUTHORS CONTRIBUTION

Both authors contributed to data management and writing of the paper.
